# Syphilis among Female Sex Workers: Results of Point-of-Care Screening during a Cross-Sectional Behavioral Survey in Burkina Faso, West Africa

**DOI:** 10.1155/2018/4790560

**Published:** 2018-11-08

**Authors:** Henri Gautier Ouedraogo, Ivlabehire Bertrand Meda, Issaka Zongo, Odette Ky-Zerbo, Ashley Grosso, Benoit Cesaire Samadoulougou, Grissoum Tarnagda, Kadari Cisse, Apoline Sondo, Nongoba Sawadogo, Yves Traoré, Nicolas Barro, Stefan Baral, Seni Kouanda

**Affiliations:** ^1^Institut de Recherche en Sciences de la Santé (IRSS/CNRST), Ouagadougou, Burkina Faso; ^2^Université Ouaga I Professeur Joseph KI-ZERBO, Ouagadougou, Burkina Faso; ^3^Institut Africain de Santé Publique (IASP), Ouagadougou, Burkina Faso; ^4^Programme d'Appui au Monde Associatif et Communautaire (PAMAC), Ouagadougou, Burkina Faso; ^5^Department of Epidemiology, Johns Hopkins Bloomberg School of Public Health, Baltimore, MD, USA; ^6^Centre Hospitalier Régional de Kaya, Kaya, Burkina Faso

## Abstract

**Background:**

Syphilis among female sex workers (FSW) remains a public health concern due to its potential impact on their health and the possibility of transmission to their clients, partners, and children. Recent data on the prevalence of syphilis in the population in West Africa are scarce. The objective of this study was to measure the seroprevalence of syphilis serological markers among female sex workers in Burkina Faso.

**Methods:**

We conducted a cross-sectional survey among FSW between February 2013 and May 2014. Participants were recruited using respondent-driven sampling (RDS) methods in five cities of Burkina Faso (Ouagadougou, Bobo-Dioulasso, Koudougou, Ouahigouya, and Tenkodogo). FSW were enrolled and screened for syphilis using a syphilis serological rapid diagnostic test. Data from all cities were analyzed with Stata version 14.0.

**Results:**

A total of 1045 FSW were screened for syphilis. Participants' mean age was 27.2 ± 0.2 years. The syphilis serological markers were detected in 5.6% (95% CI: 4.4–7.2) of the participants whereas active syphilis was seen in 1.4% (95% CI: 0.9–2.4). RDS weighted prevalence of syphilis serological markers and active syphilis by city were, respectively, estimated to be 0.0% to 11.0% (95% CI: 8.1–14.7) and 0.0% to 2.2% (95% CI: 1.1–4.4). No syphilis markers were found among Ouahigouya FSW. Low education level and high number of clients were factors associated with syphilis markers among the FSW.

**Conclusion:**

The prevalence of syphilis markers was high during this study among FSW. This highlights the need to reinforce the comprehensive preventive measures and treatment of syphilis in this population.

## 1. Background

Syphilis is a sexually transmitted disease (STD) due to *Treponema pallidum*, a spirochete bacteria belonging to the *Spirochaetaceae* family [1, 2]. The infection is often asymptomatic but can cause genital ulceration associated with enhanced risk of transmission or acquisition of HIV infection [3–7]. Syphilis remains a worldwide public health problem. Despite the availability of effective preventive measures and relatively low treatment cost, the World Health Organization (WHO) estimates that more than 10 million persons are newly infected each year [8].

A number of studies around the world have reported high prevalence of syphilis among risk groups including female sex workers (FSW) [9–12]. In Africa, the prevalence of syphilis among FSW was 2.2% in Togo [13] and between 1.5 and 89% in Sudan [14]. Studies conducted in the 1990s have reported a prevalence of 42.1% in South Africa [15] and 15% in Burkina Faso [16]. Despite this high prevalence in Africa, FSW have limited access to STIs/STDs like syphilis screening and care [17, 18]. Stigma and criminalization of sex work in some countries have complicated their access to care and have prevented the planning of efficient health systems [19–22]. FSW are identified as a population at high risk for HIV acquisition [23, 24]. Biologic risk factors for HIV include physiological factors [25] such as the presence of STDs, including syphilis [26, 27]. Recent data on syphilis prevalence in FSW are scarce in West Africa. The main objective of this study was to determine the serological prevalence and correlates of syphilis with rapid diagnostic testing among FSW in Burkina Faso.

## 2. Methods

### 2.1. Study Design

We conducted a cross-sectional survey between February 2013 and May 2014 among FSW in five cities in Burkina Faso. Participants were recruited using respondent-driven sampling (RDS) to be part of HIV seroprevalence and behavioral studies. RDS is an approach to recruit hidden and hard-to-reach populations [28].

### 2.2. Setting

The study was conducted in five cities of Burkina Faso: Ouagadougou (the capital city in the centre), Bobo-Dioulasso (in the Hauts-Bassins region), Koudougou (in the middle west), Ouahigouya (in the north), and Tenkodogo (in the middle east). These areas were selected based on the urbanization level, the prevalence of HIV, and the geographical location.

### 2.3. Study Participants

Inclusion criteria were aged ≥ 18 years, assigned female sex at birth, earned at least 50% of her income in the last 12 months from sex work, lived in the selected city during the last three months, and provided informed consent. Recruitment methods were fully described elsewhere [29]. The enrolment of FSW in each city started with an eligible seed who was enrolled and then trained to recruit other participants.

### 2.4. Sample Size

The sample size was based on expected HIV prevalence and calculated to recruit 345 FSW in the two major cities of Ouagadougou and Bobo-Dioulasso and 126 FSW in the other medium cities of Koudougou, Ouahigouya, and Tenkodogo. To calculate the sample size, we hypothesized that populations who use condoms would have 75% lower HIV prevalence than that of those not using condoms, assuming condom efficacy as 80% (73% to be cautious). A design effect of 1.5 was used associated with the RDS methods; we set 0.05 for type I errors and 80% power.

### 2.5. Data Collection

Data were collected from February to August 2013 in Ouagadougou and Bobo-Dioulasso and from February to May 2014 in Koudougou, Ouahigouya, and Tenkodogo. Upon informed consent and completion of a behavioral questionnaire, participants received counseling for HIV and syphilis screening. Participant information was anonymized and identified with a unique code number to protect confidentiality. Data collected in the questionnaire included demographic and socioeconomic information, the number of sexual partners, sexual behavior (including condom use), HIV-related knowledge, and STD screening and symptoms. In each city, one site was used for data collection. The team was trained on the study procedures and on health research ethics prior to the onset of the survey. The staff included one site manager, one coupon manager, two data collectors for questionnaire administration, a nurse for HIV and syphilis pre- and posttest counseling, and a lab technician for blood collection and sample processing for HIV and syphilis screening. Participants with positive syphilis test results were treated in situ while those with positive HIV test results were referred to dedicated staff for appropriate care.

### 2.6. Syphilis Testing

**Figure 1:**
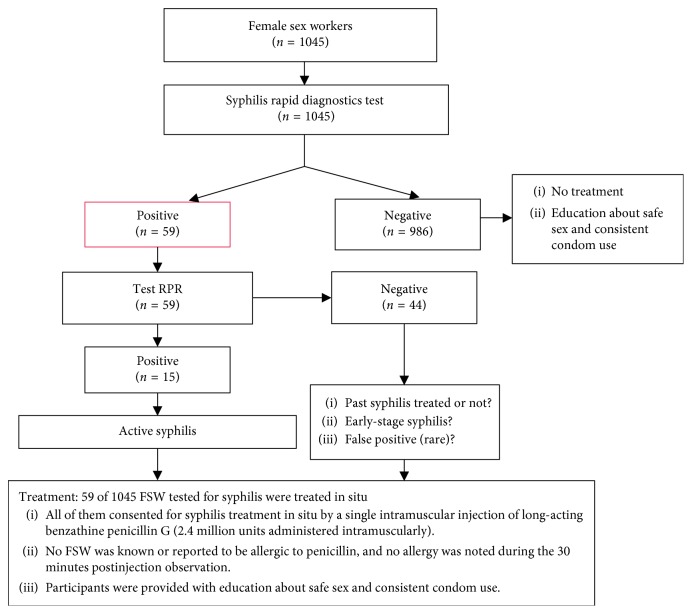
Flowchart of syphilis screening among study participants.

Figure 1 shows the flowchart of the syphilis screening among study participants. Blood samples were centrifuged, and serum obtained was used in situ for syphilis screening. *Alere Determine™ Syphilis TP* (“Alere Ltd.,” USA) rapid diagnostic test was used to screen the syphilis serological markers as per manufacturer recommendations. Positive samples were consecutively tested with a Rapid Plasma Reagin (RPR-CHARBON®) test kit (*BIOLABO SAS, French*), a nontreponemic test [30]. *Alere Determine™ Syphilis TP* is a qualitative in vitro immune-chromatographic test detecting antibodies against the *Treponema pallidum* bacteria in patient's serum, plasma, and whole blood [31]. Positivity to the *Alere Determine™ Syphilis TP* test reveals the presence of antibodies against the *Treponema pallidum* bacteria indicating the presence of either previous, acute, or treated syphilis. The nontreponemic rapid plasma reagin (RPR) test was used to determine the syphilis stage (active or not).

### 2.7. Data Entry and Statistical Analysis

Data were double entered into EpiData 3.1 (the EpiData Association, Odense, Denmark) software and analysed with Stata 14 (StataCorp, College Station, TX). RDS-weighted analysis was only used to estimate active syphilis and syphilis serological markers seroprevalence for each city. To have sufficient number of positive cases of syphilis serological markers for statistical analysis, data from all study cities were pooled. The pooled data were not analyzed using RDS weighting because there was no overlap in recruitment between study cities. Baseline survey data and biological test results of participants were summarised with descriptive statistics. Bivariate and multivariate logistic regression analyses were conducted to identify factors associated with syphilis serological markers. Associations were expressed as odds ratios (ORs) and adjusted odds ratios (aORs) with 95% confidence intervals (CIs). Results significant at the *p* < 0.2 level in the bivariate analyses were included in the multivariate analysis. Results were considered statistically significant at the *p* < 0.05 level.

### 2.8. Ethical Issues

The National Ethics Committee for Health Research in Burkina Faso reviewed and approved the study protocol prior to participant recruitment. Collected data were anonymized with a unique identifier code so that no participant could be identified by their name. All FSW detected with positive antibodies were treated in situ with one intramuscular dose of benzathine benzylpenicillin G 2.4 MUI and kept under observation over 30 minutes to capture any immediate adverse event. Positive cases presenting allergy received doxycycline 100 mg tablet over 2 weeks. FSW were sensitized on safe sex (protection measures against STDs), were encouraged to undergo a serological test for syphilis every 3 to 6 months, and were provided with condoms and lubricants free of charge.

## 3. Results

A total of 1045 FSW were screened for syphilis in the five study cities: 349 FSW in Ouagadougou, 350 in Bobo-Dioulasso, 115 in Koudougou, 102 in Ouahigouya, and 129 in Tenkodogo. Approximately 44.7% (463/1036) of those interviewed self-reported a history of STD-related symptoms during the last twelve months prior to the survey. Only 1.3% (14/1042) underwent syphilis serological testing during the last twelve months.

### 3.1. FSW Characteristics

The mean age of the study population was 27.2 ± 0.2 years; 59.8% (95% CI: 56.8–62.7) were single and 67.8% (95% CI: 64.9–70.6) came from Burkina Faso. Approximately 31.1% (95% CI: 28.4–34.0) of FSW had no formal education while more than 36% (95% CI: 33.3–39.2) had a primary school education. Unemployment outside of sex work was common among FSW, 58% (95% CI: 54.9–60.9). Only 4.7% (95% CI: 3.6–6.2) were students. The proportion of FSW who were divorced, widowed, or separated was 30.4% (95% CI: 27.7–33.3); more than 70% of the participants were mothers (Table 1).

### 3.2. Prevalence of Syphilis Serological Markers

The prevalence of syphilis serological markers was 5.6% (95% CI: 4.4–7.2) among FSW overall, and active syphilis was found among 1.4% (95% CI: 0.9–2.4) of the participants. RDS weighted prevalence of syphilis serological markers and active syphilis by city were, respectively, estimated between 0.0% and 11.0% (95% CI: 8.1–14.7) and 0.0% and 2.2% (95% CI: 1.1–4.4) (Table 2). No syphilis markers were found among FSW in Ouahigouya. As shown in Table 3, the prevalence of syphilis markers increased with age (*p*=0.025) and decreased with education level (*p* < 0.001).

Active syphilis was more prevalent among FSW with no education (2.8%) compared to those with a primary school education (0.8%) or secondary school education (0.6%) (*p*=0.037). The country of origin (Burkina Faso and other countries) was not significantly associated with the presence of serological markers among FSW.

### 3.3. Factors Associated with Syphilis Serological Markers

The factors associated with the syphilis serological markers among FSW in bivariate and multivariate analyses are presented in Table 4.

### 3.4. Bivariate Analysis

In the bivariate analysis, the risk of syphilis serological markers was 80% lower in participants with at least a secondary school education compared to FSW with no education (OR = 0.20; *p* < 0.001). Participants with at least 22 clients per week were significantly more likely to have syphilis serological markers. FSW who reported 22 to 28 clients or at least 29 clients per week were, respectively, 2.97 (OR = 2.97; *p*=0.021) and 3.34 (OR = 3.34; *p* < 0.001) times more at risk of serological marker carriage than those who reported less than 15 clients per week. FSW with a history of pregnancy were 4.31 times more likely to carry the markers compared to those who did not (OR = 4.31; *p*=0.014). Participants who sold sex for at least 6 years were more likely to carry syphilis markers than those who sold sex for less than one year (OR = 3.85; *p*=0.004). FSW in Bobo-Dioulasso had higher risk of syphilis serological marker carriage (OR = 2.87; *p*=0.001).

### 3.5. Multivariate Analysis

In multivariate analysis, after adjustment for age, education level, marital status, number of clients per week, history of pregnancy, use of condoms, and the study city, the number of clients and education level were independently associated with syphilis serological marker carriage. No education was associated with an 77% increase in the risk of syphilis serological marker carriage as compared to a secondary school level of education (aOR = 0.23; *p*=0.002). FSW having more than 29 clients per week were 2.62 times more likely to be syphilis serological marker positive compared to those with less than 15 clients per week (aOR = 2.62; *p*=0.008).

## 4. Discussion

This study found that the prevalence of syphilis serological markers and active syphilis were, respectively, 5.6% and 1.4% among FSW in Burkina Faso. The prevalence varied between study cities. To our knowledge, there are no recent data on syphilis among FSW in Burkina Faso. Overall, it appears that the prevalence of active syphilis (acute) was lower than results found in previous studies in Sub-Saharan Africa. A systematic review of congenital syphilis reported a decrease of 3.01% in syphilis prevalence between 1990 and 1999 and 1.48% between 2000 and 2009 [32]. Other studies have reported a similar reduction from 8.9% in 1993 to 1.5% in 1999 in Benin [33] and from 12.5% in 1991 to 0.8% from 1998 to 2000 in Burkina Faso [34]. This decrease of syphilis prevalence could be explained by several factors including the impact of sustained campaigns on the prevention of HIV and STDs over the last two decades in most countries [35]. This can also be explained by the introduction of syndromic management of STDs [36–38], and the widespread use of antibiotics in Africa may have contributed to the decrease. In addition to penicillin, the *Treponema pallidum* (bacterial cause of infectious syphilis) is also treated with other antibiotics such as cephalosporins (for example, cephaloridin), tetracyclins, macrolids (erythromycin and spiramycin), and chloramphenicol [2]. These antibiotics probably used for other indications have significantly contributed to reducing the incidence and evolution of infectious syphilis, including symptomatic and severe chronic forms [1].

Paradoxically, the prevalence of syphilis in our sample was increased, compared to the 2000 data [34]. This could be related to increase in sexual risk-taking, especially unprotected sex following high-active antiretroviral therapy availability and its effectiveness in HIV prevention that could lead to increase acquisition of other sexual-transmitted diseases such as syphilis [39, 40].

In addition, the prevalence of active syphilis in our study remains high in comparison with the estimate of 0.24% found in pregnant women in 2000 [41] and 1.2% in 2011 [42]. FSW are a leading group at high risk of syphilis infection due to several factors such as the multiple sexual partners and barriers to condom use [43]. Similar to prior research, in our study, FSW reporting more than 29 clients per week were three times more likely to be infected compared to those having less than 14 clients per week. Lower education was also a risk factor for syphilis infection in our study and more generally, for STDs in prior research [44, 45]. The control of STDs and particularly syphilis infection is a public health challenge [8]. Indeed, syphilis infection is very often asymptomatic and therefore untreated; thus, the potential for transmission is enhanced, and the risk of complication (including cervical cancer, higher genital tract infections, sterility, chronic pain, ectopic pregnancy, congenital abnormalities, spontaneous abortion, and associated neonatal and maternal mortality) is increased [46]. Syphilis-related genital ulcerations among FSW are important risk factors for HIV transmission [3], highlighting the relevance of early screening and treatment. In this study, all FSW who screened positive marker test were treated with a single intramuscular dose of benzathine benzylpenicillin G slow release 2.4 MUI [47] as per WHO recommendations for syphilis screening and treatment in high-risk groups, including FSW [30].

### 4.1. Limitations

In our study, the number of cases of active syphilis was low, which limited the ability to further identify factors associated with the acute infection through multivariate analysis. We instead used syphilis serological markers for analysis purposes. Furthermore, self-reporting sexual behavior in a context of social stigmatization of and discrimination toward FSW may have led to social desirability bias; however, this did not have impact on the prevalence of syphilis, as this was evaluated through syphilis serological markers. Our study provides updated data on syphilis serological markers in Burkina Faso.

## 5. Conclusion

This study provided updated epidemiological data on syphilis based on rapid diagnostic testing in situ among sex workers in Burkina Faso. We found that syphilis prevalence among FSW was relatively high and requires targeted preventive measures. Screening with rapid diagnostic tests followed by a single-dose administration of penicillin could be an effective approach to control syphilis among FSW and thus contribute to the prevention of HIV transmission. Safer sex and HIV prevention activities for FSW offer an opportunity to integrate screening and early treatment of syphilis.

## Figures and Tables

**Table 1:** Characteristics of female sex workers in Burkina Faso.

Characteristics	Number (*n*)	Proportion (%)	95% CI
*Age (years)*			
18-19	118	11.3	9.5–13.4
20–24	364	34.8	32.0–37.8
25–29	245	23.4	21.0–26.1
≥30	318	30.4	27.7–33.3
*Education level*			
None	324	31.1	28.4–34.0
Primary	377	36.2	33.3–39.2
Secondary	340	32.7	29.9–35.6
*Occupation other than sex work*			
Student	49	4.7	3.6–6.2
Worker	390	37.4	34.5–40.4
Unemployed	604	57.9	54.9–60.9
*Marital status*			
Single	625	59.8	56.8–62.7
Married or cohabitating	102	9.8	8.1–11.7
Divorced/separated/widowed	318	30.4	27.7–33.3
*Number of biological children*			
0	277	26.5	23.9–29.3
1	397	38.0	35.1–41.0
2+	371	35.5	32.7–38.4
*Duration in sex work*			
<1 year	237	23.0	20.5–25.7
1–5 years	506	49.2	46.1–52.2
6+ years	286	27.8	25.1–30.6
*Number of clients per week*			
1–14	451	45.9	42.8–49.1
15–21	241	24.5	21.9–27.3
22–28	71	7.2	05.7–09.0
29–80	219	22.3	19.8–25.0
*Has been pregnant?*			
No	188	18.0	15.8–20.5
Yes	856	82.0	79.5–84.2
*Past 12 months consistent condom use*			
No	403	39.5	36.5–42.5
Yes	618	605.	57.5–63.5
*Past 12 months STD symptoms*			
No	573	55.3	52.1–58.1
Yes	463	44.7	41.5–47.6
*Past 12 months STD screening*			
No	829	79.6	77.0–81.9
Yes	213	20.4	18.1–23.0
*HIV status*			
Positive	168	16.1	13.9–18.4
Negative	877	83.9	81.7–86.0
*Syphilis serological marker status*			
Positive	59	5.6	4.4–7.2
Negative	986	94.4	92.8–95.6
*Active syphilis status*			
Positive	15	1.4	0.9–2.4
Negative	1030	98.6	97.6–99.1
*Study site*			
Ouagadougou	349	33.4	30.6–36.3
Bobo-Dioulasso	350	33.5	30.9–36.4
Koudougou	115	11.0	9.2–13.0
Ouahigouya	102	9.8	8.1–11.7
Tenkodogo	129	12.3	10.5–14.5
*Country of origin*			
Burkina Faso	709	67.8	64.9–70.6
Other countries	336	32.2	29.4–35.0
*Overall*	1045	100.0	

STD = sexually transmitted disease; CI = confidence interval.

**Table 2:** Active syphilis and syphilis serological markers prevalence among female sex workers by city.

Variables	All cities (*n*=1045)	Ouagadougou (*n*=349)		Bobo-Dioulasso (*n*=350)		Koudougou (*n*=115)		Ouahigouya (*n*=102)		Tenkodogo (*n*=129)	
	**%**	Unadjusted %	RDS-adjusted % (95% CI)	Unadjusted %	RDS-adjusted % (95% CI)	Unadjusted %	RDS-adjusted % (95% CI)	Unadjusted %	RDS-adjusted % (95% CI)	Unadjusted %	RDS-adjusted % (95% CI)
Syphilis serological markers	**5.6**	4.3	4.4 (2.7–7.2)	**11.4**	11.0 (8.1–14.7)	**1.7**	1.7 (0.4–6.6)	**0.0**	—	**1.6**	1.5 (4.4–7.2)
Active syphilis	**1.4**	1.4	1.4 (0.6–3.3)	**2.3**	2.2 (1.1–4.4)	**1.7**	1.7 (0.4–6.6)	**0.0**	—	**0.0**	—

**Table 3:** Prevalence of syphilis serological markers and of active syphilis among female sex workers in Burkina Faso.

FSW characteristics	*n*	Syphilis serological markers (%)	*p*	Active syphilis (%)	*p*
*Age (years)*					
18-19	118	3.4	0.025^*∗*^	0.0	0.558
20–24	364	3.8		1.6	
25–29	245	5.3		1.2	
≥30	318	8.8		1.9	
**All ages**	**1045**	**5.6**		**1.4**	
*Education level*					
None	324	9.3	<0.001^*∗∗∗*^	2.8	0.037^*∗*^
Primary	377	5.4		0.8	
Secondary	340	2.1		0.6	
*Occupation other than sex work*					
Student	49	8.2	0.216	2.0	0.723
Worker	390	4.2		1.3	
Unemployed	604	5.7		1.5	
*Marital status*					
Single	625	4.5	0.133	0.8	0.038^*∗*^
Married or cohabitating	102	6.9		1.0	
Divorced/separated/widowed	318	7.5		2.8	
*Number of biological children*					
0	277	26.5	0.005	0.36	0.162
1	397	38.0		1.52	
2+	371	35.5		2.16	
*Duration in sex work*					
<1 year	237	2.5	0.005^*∗∗*^	0.4	0.169
1–5 years	506	5.3		1.4	
6+ years	286	9.1		2.5	
*Number of clients per week*					
1–14	451	3.5	0.001^*∗∗∗*^	0.9	0.014^*∗*^
15–21	241	4.6		0.4	
22–28	71	9.9		2.8	
29–80	219	11.0		3.7	
*Has been pregnant*					
No	188	1.6	0.008^*∗∗*^	0.5	0.494
Yes	856	6.5		1.6	
*Past 12 months consistent condom use*					
No	403	6.9	0.124	1.2	0.418
Yes	618	5.0		1.6	
*Past 12 months STD symptoms*					
No	573	5.2	0.876	1.6	0.806
Yes	463	6.3		1.3	
*Past 12 months STD screening*					
No	829	6.1	0.177	1.8	0.051
Yes	213	3.8		0.0	
*HIV status*					
Positive	168	7.1	0.359	0.6	0.488
Negative	877	5.4		1.6	
*Country of origin*					
Burkina Faso	709	5.9	0.572	1.4	1.000
Other countries	336	5.1		1.5	

STD = sexually transmitted disease; ^*∗*^*p* < 0.05, ^*∗∗*^*p* < 0.01, and ^*∗∗∗*^*p* < 0.001.

**Table 4:** Factors associated with syphilis serological markers among female sex workers in Burkina Faso.

Characteristics	FSW (*n*)	Syphilis serological markers (%)	Bivariate analysis			Multivariate analysis		
			OR	95% CI	*p*	aOR	95% CI	*p*
*Age (years)*								
18-19	118	3.4	1	—	—	—	—	—
20–24	364	3.8	1.14	0.36–3.53	0.820	—	—	—
25–29	245	5.3	1.59	0.50–5.00	0.422	—	—	—
≥30	318	8.8	2.75	0.94–8.02	0.064	1.18	0.30–4.63	0.805
All ages	1045	5.6						
*Education level*								
None	324	9.3	1	—	—	—	—	—
Primary	377	5.6	0.57	0.32–1.03	0.063	0.62	0.33–1.13	0.085
Secondary	340	2.1	0.20	0.09–0.47	<0.001	0.23	0.09–0. 60	0.002
*Occupation other than sex work*								
Student	49	8.2	1	—	—	—	—	—
Worker	390	4.1	0.48	0.15–1.50	0.208	—	—	—
Unemployed	604	5.7	0.77	0.26–2.27	0.644	—	—	—
*Marital status*								
Single	625	4.5	1	—	—	—	—	—
Married or cohabitating	102	6.9	1.57	0.66–3.69	0.301	—	—	—
Divorced/separated/widowed	318	7.5	1.74	0.99–3.05	0.054	0.83	0.40–1.72	0.631
*Number of biological children*								
0	277	2.17	1					
1	397	5.79	2.78	1.11–6.91	0.028	1.69	0.56–5.14	0.351
2+	371	8.09	3.97	1.63–9.68	0.002	1.26	0.38–4.15	0.706
*Duration in sex work*								
<1 year	237	2.5	1	—	—	—	—	—
1–5 years	506	5.3	2.17	0.88–5.32	0.091	1.04	0.37–2.92	0.933
6+ years	286	9.1	3.85	1.56–9.51	0.004	1.12	0.38–3.31	0.833
*Number of clients per week*								
1–14	451	3.5	1	—	—	—	—	—
15–21	241	4.6	1.30	0.59–2.84	0.512			
22–28	71	9.9	2.97	1.18–7.50	0.021	1.97	0.73–5.26	0.177
29–80	219	11.0	3.34	1.73–6.44	<0.001	2.62	1.28–5.35	0.008
*Has been pregnant*								
No	188	1.6	1	—	—	—	—	—
Yes	856	6.5	4.31	1.33–3.94	0.014	1.68	0.38–7.38	0.493
*Past 12 months consistent condom use*								
No	403	6.9	1	—	—	—	—	—
Yes	618	5.0	0.71	0.41–1.19	0.190	0.65	0.35–1.19	0.163
*Past 12 months STD symptoms*								
No	573	5.2	1					
Yes	463	6.3	1.21	0.71–2.04	0.710	—	—	—
*Past 12 months STD screening*								
No	829	6.1	1	—	—	—	—	—
Yes	213	3.8	1.20	0.71–2.04	0.478	—	—	—
*HIV status*								
Negative	877	5.4	1					
Positive	168	7.1	1.35	0.70–2.61	0.360	—	—	—
*Study site*								
Ouagadougou	349	4.3	1					
Bobo-Dioulasso	350	11.4	2.87	1.56–5.30	0.001	1.74	0.85–3.56	0.130
Koudougou	115	1.7	0.39	0.09–1.74	0.221	0.40	0.09–1.88	0.248
Ouahigouya	102	0.0	—			—		
Tenkodogo	129	1.5	0.35	0.08–1.55	0.168	0.19	0.02–1.53	0.120
*Country of origin*								
Burkina Faso	709	5.9	1	—	—	—	—	—
Other countries	336	5.1	0.82	0.47–1.46	0.515	—	—	—

STD = sexually transmitted disease; OR = odds ratio; aOR = adjusted odds ratio; CI = confidence interval.

## Data Availability

The datasets used during the current study are available from the corresponding author on reasonable request.

## References

[1] Hook E. W. (2017). Syphilis. *The Lancet*.

[2] Singh A. E., Romanowski B. (1999). Syphilis: review with emphasis on clinical, epidemiologic, and some biologic features. *Clinical Microbiology Reviews*.

[3] Greenblatt R. M., Lukehart S. A., Plummer F. A. (1988). Genital ulceration as a risk factor for human immunodeficiency virus infection. *AIDS*.

[4] Syphilis and HIV, March 2017, http://hivinsite.ucsf.edu/InSite?page=kb-00&doc=kb-05-01-04

[5] Salado-Rasmussen K. (2015). Syphilis and HIV co-infection. Epidemiology, treatment and molecular typing of *Treponema pallidum*. *Danish Medical Journal*.

[6] Stevenson J., Heath M. (2006). Syphilis and HIV infection: an update. *Dermatologic Clinics*.

[7] Zetola N. M., Klausner J. D. (2007). Syphilis and HIV infection: an update. *Clinical Infectious Diseases*.

[8] World Health Organization (2012). *Prevention and Treatment of HIV and other Sexually Transmitted Infections for Sex Workers in Low- and Middle-Income Countries: Recommendations for a Public Health Approach*.

[9] Kang D., Liao M., Jiang Z. (2011). Commercial sex venues, syphilis and methamphetamine use among female sex workers. *AIDS Care*.

[10] Zhou C., Rou K., Dong W. M. (2014). High prevalence of HIV and syphilis and associated factors among low-fee female sex workers in mainland China: a cross-sectional study. *BMC Infectious Diseases*.

[11] Tao X. H., Jiang T., Shao D. (2014). High prevalence of syphilis among street-based female sex workers in Nanchang, China. *Indian Dermatology Online Journal*.

[12] Kakchapati S., Singh D. R., Rawal B. B., Lim A. (2017). Sexual risk behaviors, HIV, and syphilis among female sex workers in Nepal. *HIV/AIDS-Research and Palliative Care*.

[13] Halatoko W. A., Landoh D. E., Saka B. (2017). Prevalence of syphilis among female sex workers and their clients in Togo in 2011. *BMC Public Health*.

[14] Elhadi M., Elbadawi A., Abdelrahman S. (2013). Integrated bio-behavioural HIV surveillance surveys among female sex workers in Sudan, 2011-2012. *Sexually Transmitted Infections*.

[15] Ramjee G., Karim S. S., Sturm A. W. (1998). Sexually transmitted infections among sex workers in KwaZulu-Natal, South Africa. *Sexually Transmitted Diseases*.

[16] Nicolas S. L., Sangare M. L., Compaore I. P. (1998). Prevalence and risk of HIV infection among female sex workers in Burkina Faso. *International Journal of STD & AIDS*.

[17] Scorgie F., Nakato D., Harper E. (2013). ‘We are despised in the hospitals’: sex workers’ experiences of accessing health care in four African countries. *Culture, Health & Sexuality*.

[18] Wanyenze R. K., Musinguzi G., Kiguli J. (2017). “When they know that you are a sex worker, you will be the last person to be treated”: perceptions and experiences of female sex workers in accessing HIV services in Uganda. *BMC International Health and Human Rights*.

[19] Kim H.-Y., Grosso A., Ky-Zerbo O. (2018). Stigma as a barrier to health care utilization among female sex workers and men who have sex with men in Burkina Faso. *Annals of Epidemiology*.

[20] Davis A., Meyerson B. E., Aghaulor B. (2016). Barriers to health service access among female migrant Ugandan sex workers in Guangzhou, China. *International Journal for Equity in Health*.

[21] Lafort Y., Lessitala F., Candrinho B. (2016). Barriers to HIV and sexual and reproductive health care for female sex workers in Tete, Mozambique: results from a cross-sectional survey and focus group discussions. *BMC Public Health*.

[22] Wahed T., Alam A., Sultana S. (2017). Barriers to sexual and reproductive healthcare services as experienced by female sex workers and service providers in Dhaka city, Bangladesh. *PLoS One*.

[23] Baral S., Beyrer C., Muessig K. (2012). Burden of HIV among female sex workers in low-income and middle-income countries: a systematic review and meta-analysis. *The Lancet Infectious Diseases*.

[24] Baral S., Ketende S., Green J. L. (2014). Reconceptualizing the HIV epidemiology and prevention needs of female sex workers (FSW) in Swaziland. *PLoS One*.

[25] Kaushic C., Roth K. L., Anipindi V., Xiu F. (2011). Increased prevalence of sexually transmitted viral infections in women: the role of female sex hormones in regulating susceptibility and immune responses. *Journal of Reproductive Immunology*.

[26] HIV Prevention through Early Detection and Treatment of Other Sexually Transmitted Diseases—United States Recommendations of the Advisory Committee for HIV and STD Prevention, March 2017, https://www.cdc.gov/mmwr/preview/mmwrhtml/00054174.htm 9701544

[27] Syphilis and HIV: the intersection of two epidemics, NEJM Journal Watch, http://www.jwatch.org/ac201009030000001/2010/09/03/syphilis-and-hiv-intersection-two-epidemics

[28] Heckathorn Douglas D. (1997). Respondent-driven sampling: a new approach to the study of hidden populations. *Social Problems*.

[29] Ouedraogo H. G., Ky-Zerbo O., Baguiya A. (2017). HIV among female sex workers in five cities in Burkina Faso: a cross-sectional baseline survey to inform HIV/AIDS Programs. *AIDS Research and Treatment*.

[30] World Health Organisation/TDR (2006). *The Use of Rapid Syphilis Test*.

[31] Bocoum F., Ouedraogo H., Tarnagda G. (2015). Evaluation of the diagnostic performance and operational characteristics of four rapid immunochromatographic syphilis tests in Burkina Faso. *African Health Sciences*.

[32] Kenyon C. R., Osbak K., Tsoumanis A. (2016). The global epidemiology of syphilis in the past century—a systematic review based on antenatal syphilis prevalence. *PLoS Neglected Tropical Diseases*.

[33] Alary M., Mukenge-Tshibaka L., Bernier F. (2002). Decline in the prevalence of HIV and sexually transmitted diseases among female sex workers in Cotonou, Benin, 1993-1999. *AIDS*.

[34] Nagot N., Meda N., Ouangre A. (2004). Review of STI and HIV epidemiological data from 1990 to 2001 in urban Burkina Faso: implications for STI and HIV control. *Sexually Transmitted Infections*.

[35] Ghys P. D., Diallo M. O., Ettiègne-Traoré V. (2002). Increase in condom use and decline in HIV and sexually transmitted diseases among female sex workers in Abidjan, Côte d’Ivoire, 1991–1998. *AIDS*.

[36] Vuylsteke B. (2004). Current status of syndromic management of sexually transmitted infections in developing countries. *Sexually Transmitted Infections*.

[37] Johnson L. F., Dorrington R. E., Bradshaw D., Coetzee D. J. (2011). The effect of syndromic management interventions on the prevalence of sexually transmitted infections in South Africa. *Sexual & Reproductive Healthcare*.

[38] Kenyon C. R., Osbak K., Chico R. M. (2014). What underpins the decline in syphilis in Southern and Eastern Africa? An exploratory ecological analysis. *International Journal of Infectious Diseases*.

[39] Scheer S., Chu P. L., Klausner J. D., Katz M. H., Schwarcz S. K. (2001). Effect of highly active antiretroviral therapy on diagnoses of sexually transmitted diseases in people with AIDS. *The Lancet*.

[40] Rekart M. L., Ndifon W., Brunham R. C. (2017). A double-edged sword: does highly active antiretroviral therapy contribute to syphilis incidence by impairing immunity to *Treponema pallidum*?. *Sexually Transmitted Infections*.

[41] Sombie I., Meda N., Cartoux M. (2000). Seroprevalence of syphilis among women attending urban antenatal clinics in Burkina Faso, 1995–8. *Sexually Transmitted Infections*.

[42] Kirakoya-Samadoulougou F., Defer M.-C., Yaro S. (2010). Low seroprevalence of syphilis in Burkina Faso. *Sexually Transmitted Infections*.

[43] Cai R., Tan J. G., Chen L., Richardus J. H., de Vlas S. J. (2013). Prevalence and risk factors of syphilis infection among female sex workers in Shenzhen, China: an observational study (2009–2012). *Tropical Medicine & International Health*.

[44] Solomon M. M., Smith M. J., Del Rio C. (2008). Low educational level: a risk factor for sexually transmitted infections among commercial sex workers in Quito, Ecuador. *International Journal of STD & AIDS*.

[45] Celentano D. D., Nelson K. E., Suprasert S. (1996). Epidemiologic risk factors for incident sexually transmitted diseases in young Thai men. *Sexually Transmitted Diseases*.

[46] Newman L., Kamb M., Hawkes S. (2013). Global estimates of syphilis in pregnancy and associated adverse outcomes: analysis of multinational antenatal surveillance data. *PLoS Medicine*.

[47] Clement M. E., Okeke N. L., Hicks C. B. (2014). Treatment of syphilis: a systematic review. *JAMA*.

